# Mapping the spatial variability of subsurface resistivity by using vertical electrical sounding data and geostatistical analysis at Borena Area, Ethiopia

**DOI:** 10.1016/j.mex.2022.101792

**Published:** 2022-07-25

**Authors:** Yahya Ali Abdulkadir, Shimeles Fisseha

**Affiliations:** a School of Earth sciences, Addis Ababa University, Ethiopia; b Department of Physics, University of Gondar, Ethiopia; c Institute of Geophysics, Space Science and Astronomy, Addis Ababa University, Ethiopia

**Keywords:** Vertical electrical sounding, Geostatistics, Approximate interpretation, Borena, Southern main Ethiopia rift (SMER)

## Abstract

Vertical electrical sounding survey has been done to map and visualize resistivity distribution at Borena basin. The area is situated in Southern Main Ethiopian Rift, where Rirriba rift, Mega rift and associated fractures define the structural setting of the area. It is covered by Quaternary deposits, Quaternary and Tertiary basaltic rocks and Precambrian metamorphic rocks. Bullal basaltic formation outstretch in the Rirriba rift and it is thought to be potential groundwater aquifer. About 288 Vertical Electrical Sounding data were collected. Inflection and extreme points were used to identify characteristic points. Variograms are modeled and kriging interpolation is used to map distribution of resistivity, determined from characteristic points, in the area. Very low to low resistivity variations are mapped in northern end of the study area, whereas medium to moderately resistive ground are mapped in the middle and southern part of the area. The low resistivity horizon at the shallow subsurface could be due to salinity since the area occupy numerous saline craters and maars. Approximate mapping of large sets of Vertical Electrical Sounding data with geostatistical treatment has facilitated the interpretation and provided a sound picture of the subsurface.

• Inflection and extreme points were extracted from smothed VES curves to identify characteristic points.

• Variograms are modeled and kriging interpolation is used to map 3D distribution of resistivity data.

## Graphical abstract

**Figure:**
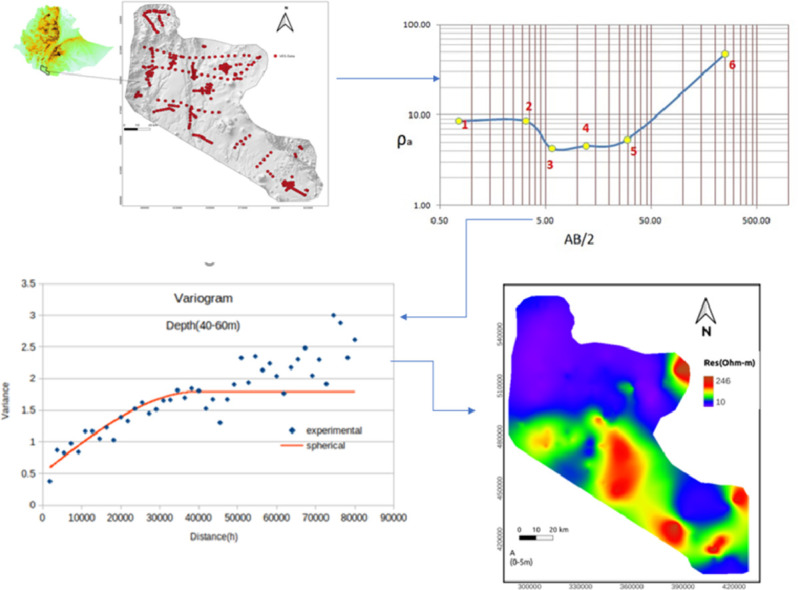

Specifications table

**Table:** 

Subject Area;	Earth and Planetary Sciences
More specific subject area;	*Hydrogeophysics*
Method name;	*Electrical Resistivity Method*
Name and reference of original method;	Santana, J. M., & Medeiros, W. E. (2016). Automatic approximate mapping of the subsurface resistivity from apparent resistivity data using geostatistics. *Geophysics, 81*(2), E177–E186. https://doi.org/10.1190/GEO2015-0189.1
Resource availability;	*All resources included in the article*

## Introduction

There are various approaches to measuring the resistivity of the subsurface material. Four electrode arrays are commonly employed in subsurface resource exploration, particularly in groundwater and environmental studies. Because rocks resistivity mainly depends on porosity and water content/saturation, geoelectrical methods are essential in hydrogeological problems. Direct current resistivity (DC) is the most commonly used technique to determine the resistivity of the subsurface materials of the earth. In groundwater exploration, the classical Schlumberger array is most widely exploited due to its field ease and deeper penetration depth. In the Schlumberger array the apparent resistivity is calculated from the employed current, recorded potential and geometry of electrode positions. The apparent resistivity is a function of the true resistivity and thickness of layers. In homogeneous strata, the apparent resistivity approximates the true resistivity very well. However, in the heterogeneous ground the apparent resistivity has to be inverted to get true resistivity of the ground. Using master curves by Orellana & Mooney [25], Curve matching is commonly used to determine initial models. These models are interpreted using inversion techniques and inaccuracies are minimized by fitting field curves and theoretical curves. However, different input models can produce the same output giving ambiguity. Even the best-fitting model curve can only provide a good estimate of the true resistivity. Auxiliary information from other geophysical results or geological background is necessary to distinguish the correct models. In this study, a methodology by Santana & Medeiros [28] is extended for large sets of vertical electrical sounding data with geostatistical treatment. The paper focuses on the approximate mapping of Vertical Electrical Sounding (VES) data collected to better understand the resistivity distribution using a simple characterization of VES curves collected at the Borena area.

**Fig. 1:**
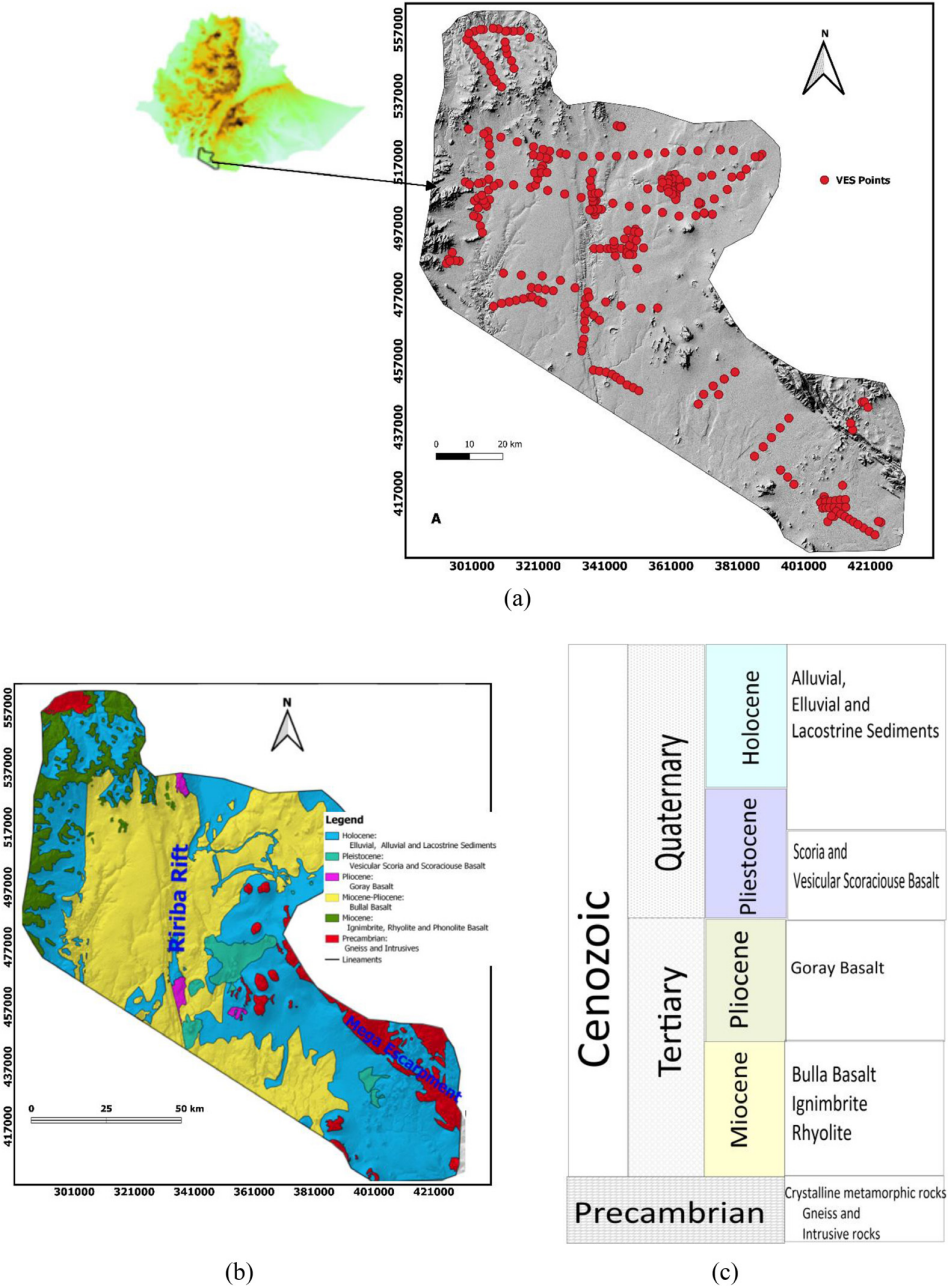
Location and Geological maps of the study area (a) Location map: Ethiopia(left), Digital Elevation Model with VES data distribution of the study area (right), (b) Geological map of the area with main age groups and (c) Simplified stratigraphy of rocks in the study area.

The study area, Borena, is located in the southern sector of Ethiopia, bordering on Kenya. Geographically the site is bound to UTM x- coordinates of 285261, 399276, and Y-coordinates of 436768, 562739m (Fig. 1a). The region is explained as arid to semi-arid climate with heterogeneous geologic environments.

**Geological setting**

Recently, OWWDSE [26], Daddi [6], Fanta [11], MAB [24], Deyassa [8], Kebede [20], [22] studied the geological environments of the region for various purposes. Basement hills and upland plains, volcanic mountains and highland plateaus, volcanic plains and lowland areas, recent volcanic landforms, Mega rift escarpment and Rirriba fault zone, define the geomorphology of the Borena area [26]. The fluvial environments are described by volcanic plains, lowland areas and Rirriba fault zones. At the same time, the basement hills, upland plains, Volcanics Mountains, highland plateau and Mega rift escarpment from denuded landforms.

According to Kazmin [18,19] and Tefera and Cherinet [31], Precambrian basement, Mesozoic sediment, Cenozoic volcanic and sediments are mapped in the study area. Chronostratigraphic units occupying southern Ethiopia, where the study area is situated, are Precambrian crystalline basement, Mesozoic sediments, Cenozoic volcanic with sporadic sediments and superficial deposits [18,^19^,31]. The major time-stratigraphic units known in the region are Quaternary, Tertiary and Precambrian basement rocks. Holocene deposits such as alluvial, eluvial and lacustrine sediments and Miocene-pliocene rocks such as Goray basalt, Bullal basalt, ignimbrite and rhyolite rocks cover most of the study area, particularly geoelectrical studies were conducted in these formations. The Precambrian strata in the region consist of crystalline rocks and associated intrusives, and Tertiary to Quaternary strata consists of volcano-sedimentary rocks, where the superficial deposits are all grouped to Quaternary age (thought to be formed in the Holocene period) [10,26]. The older metamorphic complexes of the Precambrian crystalline basements comprise high-grade gneisses, schists and granulites, intruded locally by sets of plutonic rocks, weakly to moderately metamorphosed sedimentary, basic volcanic and mafic-ultramafic rocks [24]. NW-SE trending Mega Rift, N-S stretching Rirriba Rift, along which the Rirriba River flows, and Volcanic activities like craters (maars) and cones represent the structural setting of the study area [3,^10^,21].

## Methodology

### Field data

The Oromia Water Works Supervision and Designe Enterprise (OWWSDE) led the acquisition of the vertical electrical sounding data in three phases for Borena ground water project. The apparent resistivity data were collected at Borena area of Oromia regional state, southern Ethiopia. The Shlumberger array has been employed for the VES survey. The survey lines and the sounding points have been selected so as to evenly cover the interesting portions of the plains and transect possible structural features (Fig. 1a). A maximum half-current electrode separation (AB/2) of 1000 m was used to map the subsurface to the depths of potential water bearing horizons.

The data processing and interpretation methodology is based on the following simpleprocedures:

• Smoothing the VES curve which helps minimize errors,
• Identify Characteristics Points (CPs) from the smoothed VES curve,
• Obtain sparse point estimates from the CPs such as resistivities and depths,
• Interpolate these points using kriging by modeling semi-variograms.

### Resistivity approximation

A methodology for approximate mapping of resistivity of VES data data in 2-dimension using geostatistical approaches has been presented by Santana & Medeiros [28].

The method is based on subsurface sparse point resistivity estimates obtained from inflection and extreme points of VES curves. Inflection and extreme points are essential to describe the morphological characteristics of a function. It can be used to detect points where the polarity of curves changes.

For a layered, nearly horizontal and laterally homogeneous earth, 1D approximation often yields reliable results. However, this approximation does not provide complete information for complex structures and heterogeneous earth. Therefore, 2D and 3D approximation of VES data are important to get full and reliable picture of the subsurface under investigation. For sparse data covering large areas, interpolation is a common undertaking to estimate values where actual data is not collected (non-data points). Predicting values of non-data points from sparse information is as essential as collected data points. Therefore, care should be given to choosing an objective interpolation method that offers spatial correlation. Geostatistics has long been used for problems with spatial and temporal data. It has been used for geophysical methods, particularly in electrical resistivity data, to improve geological interpretation [17]. In the following, characteristics points (CPs) of apparent resistivity data obtained from inflection and extreme points are geostatistically treated to map the 3D representation of the subsurface resistivity. In this approach, subsurface spares points of resistivity estimates obtained from inflection and extreme points of VES data are used to compose appropriate semi-variograms for kringing.

### Depth approximation

The depths of the sparse points obtained are assigned to approximate the depth of investigation of VES data. The depth of investigation for resistivity method depends on the maximum electrode separation and partly on the resistivity difference of layers. There is no simple rule of thumb for determining depth of investigation for electrical resistivity methods because of a huge range of possible electrical structures and a variety of arrays, but half the current electrode separation can be used as a guide to the maximum depth of penetration. The depth of penetration of the electrical resistivity method is mainly dependent on the length of the separation distance between current electrodes and potential electrodes and the resistivity contrast of layers.

A large separation of current electrodes in a Schlumberger configuration can obtain a deeper current penetration of current. More representative potential for deeper layers can also be measured when current and potential electrodes separation is large enough. The depth of investigation for VES is commonly taken between 0.1 and 0.3 times the length of separation of current electrodes (AB). According to Fröhlich [12] the depth of current penetration is about 1/4 to 1/3 of the separation of current electrodes (AB). In the Schlumberger array, when the separation of potential and current electrodes are far apart, the resistivity represents the subsurface condition well [2]. According to Edwards [9] depth of penetration of the current is directly proportional to the total electrode spacing. Barker [1] explained the dependence of depth of investigation with the relative positions of both current and potential electrodes. Sharma [30] showed the depth of investigation depends on the current source, the sensitivity of the array type to near-surface inhomogeneities, resistivity contrast between subsurface layers and anisotropic electrical conditions. Therefore, in this study, one-fourth of the current electrode separation is used to approximate the depth of penetration for all VES data collected in the area.

### Identifying characteristics points (CPs) from sounding curves

**Fig. 2:**
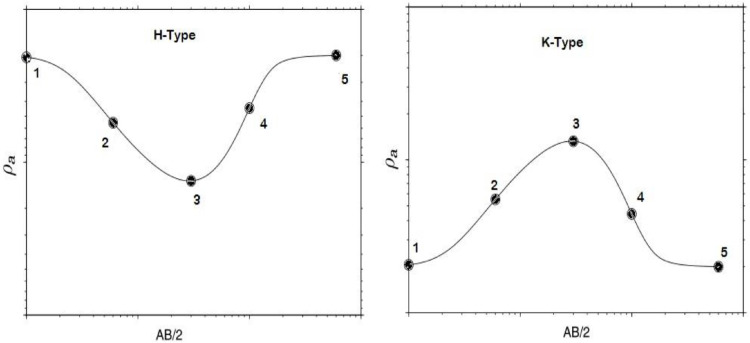
Typical H and K Schlumberger sounding curves and its CPs (marked as circles) [28]. CPs are inflection points, extreme (local minima and maxima) points and end points of a sounding curve numbered 1-5.

Characteristics Points (CPs) can be obtained from resistivity curves using first and second derivatives. Points where a function changes in concavity (inflection points) are similar to critical points in the first derivative; inflection points can also occur at the zero of the second derivative. Maxima and minima of a curve can also be determined using the second derivative of curves (functions). In this study, a python code is written to smooth curves and find CPs from each sounding data. Fig. (2) shows how the characteristic points are determined.

Depths to CPs can be assigned by using 1/4 of the electrode spacing of the corresponding CPs. In contrast, resistivities are assigned apparent resistivity value of the previous CP except for the starting and ending point CPs apparent resistivity of same CPs are assigned (eg. for CP-1 and CP-5, ρ(1) and ρ(5) are assigned. For CP-2, CP-3 and CP-4, resistivity values of *ρ*(1), *ρ*(2), and *ρ*(3) are given, respectively).

### Geostatistical modeling

Geostatistics is a statistical method focusing on spatial or spatio-temporal datasets. The geostatistical analysis incorporates spatial coordinates of the data within the analysis. It is applied in various environmental and geoscientific fields, including hydrogeology (eg. [23]), geochemistry (eg. [27]), geophysics (eg. [4]), hydrology (eg. [14]), soil science (eg. [15]) and environmental analysis (eg. [7]).

#### Variogram

The primary work in geostatistical modeling is exploring the characteristics of the data (which is called Exploratory Data Analysis (EDA)). Treatment of outliers and decisions on the transformation of data is made after exploratory data analysis. Histograms and box-and-whisker plots are simple ways to summarize statistical analysis such as Minimum, Quartiles, Maximum, Mean, Median and Outliers. After exploratory data analysis has been done, the next step in the geostatistical analysis is modeling variogram. The variogram is the classical way of measuring spatial correlation in geostatistics. It is a geostatistical tool used to measure the variability in geographically distributed data.

The essential concept in geostatistical modeling is that spatial observations close to each other are more alike than observations far apart. The experimental variogram measures dissimilarities between non sampled values and a nearby data value. Thus, the autocorrelation at various distances called lag (h) is calculated. The experimental variogram *γ* (semivariogram) is half the average squared difference between the values at *Z*(*x_i_*) and *Z*(*x_i_* + *h*)

(1) $$\gamma \left(h\right)=\frac{1}{2N\left(h\right)}\sum_{i=1}^{N\left(H\right)}{\left(Z\left(xi\right)\;-\;Z\left(xi\;+\;h\right)\right)}^{2}$$

Where *N*(*h*) is the number of data pairs within a given class of distance and direction. *γ* is small if values separated by a given lag (h) are autocorrelated, whereas it gets large if the dissimilar values. Experimental variograms were acquired by calculating variogram at different lags, and a standard variogram is fitted to determine variogram parameters such as *sill, range* and the *nugget* effect. The commonly used standard variogram models are given in equations (2 – 5)

• The Spherical model

(2) $$\begin{array}{c} \gamma \left(h\right)=\left\{ \begin{array}{c} c_{0}+\left\{\frac{3h}{2r}-\frac{1}{2}{\left(\frac{h}{r}\right)}^{3}\right\}\;\mathrm{for}\;0<h\leq r\\ c_{0}+c\;\mathrm{for}\;h>r\\ 0\;\mathrm{for}\;h=0 \end{array}\;\right. \end{array}$$

Here *c* is the spatially correlated variance, and *r* is the range. The quantity *c*_0_ + *c* is known as the sill.

• The Gaussian model

(3) $$\gamma \left(h\right)=\left\{ \begin{array}{c} c_{0}+c\;\left\{1-\exp\left(-\frac{h^{2}}{r^{2}}\right)\right\}\;for\;0<h\\ 0\;for\;h=0 \end{array}\right.$$

*• The Exponential model*

(4) $$\gamma \left(h\right)=\;\left\{ \begin{array}{c} c_{0}+c\;\left\{1-\exp\left(-\frac{h}{r}\right)\right\}\;for\;0<h\\ 0\;for\;h=0 \end{array}\right.$$

• The Power model

(5) $$\gamma \left(h\right)=\left\{ \begin{array}{c} c_{0}+bh{\;}^{n}\;for\;0<h\\ 0\;for\;h=0 \end{array}\right.$$

The calculated experimental Variogram is fitted to the standard variogram models such as Spherical, Exponential, Gaussian, Power etc. [15,16]. Iterative reweighted least squares estimation fit the experimental variogram to the standard variogram models. The weights are determined based on the number of point pairs or distance. Most commonly, the weights are determined using *N_j_/h*^2^*_j_*, where *N_j_* is the number of pairs at a certain lag, and *h_j_* is the distance (lag).

Various models of variogram may fit the experimental variogram. The choice of variogram model among them is based on the root mean square error (RMSE) values of the candidate models with the experimental semi-variogram given by (6). The variogram with the smallest RMSE is chosen as the suitable model fitting the data.

(6) $$RMSE\left(\%\right)=\sqrt{\frac{1}{n}\sum_{i}^{n}{\left(Z\left(x_{i}\right)-Z^{*}\left(x_{i}\right)\right)}^{2}}*100$$

The spatial variability characteristics of the modeled variogram has to be measured before applying the variogram. The Nugget to Sill ratio of the model variogram represents the spatial correlation. The nugget value represents short-range variability, whereas the sill value represents the overall variability inside the variables. For an acceptable variogram, the nugget to sill ratio must be between 0 and 1 when this ration is *<* 0*.*25, the variable is firmly spatially dependent; when the ratio lies between 0*.*25 to 0*.*75, then the variable is considered moderately spatially dependent, while the nugget to sill ratio is *>* 0*.*75, the variable is considered weakly spatially dependent [13,32]. Table (3) shows the spatial correlation of variogram models used in the study area. Once the suitable variogram model is chosen and parameters assigned, the next step in geostatistical modeling is applying kriging interpolation using parameters obtained from the selected Variogram.

#### Kriging

Kriging is one of the robust interpolation techniques accounting the spatial continuity of variables. It integrates both the spatial correlation and the dependence in the prediction of a known variable. It is the best interpolation technique/estimator for spatially varying data [29].

Spatial continuity is a concept that involves small values of an attribute are in geographic proximity to other small values, while high values are close to other high values. The resistivity data in this study are distributed sparsely, covering a large area. The approximate sampling distance for VES data is 1-5km.

Interpolation of these data set requires considerable effort and a good choice of estimator. Kriging is an unbiased linear interpolation technique. It uses a weighted average of neighboring samples to estimate the unknown values at a particular location. The weights are determined using variogram models.

## Results

### VES data at Borena

**Fig. 3:**
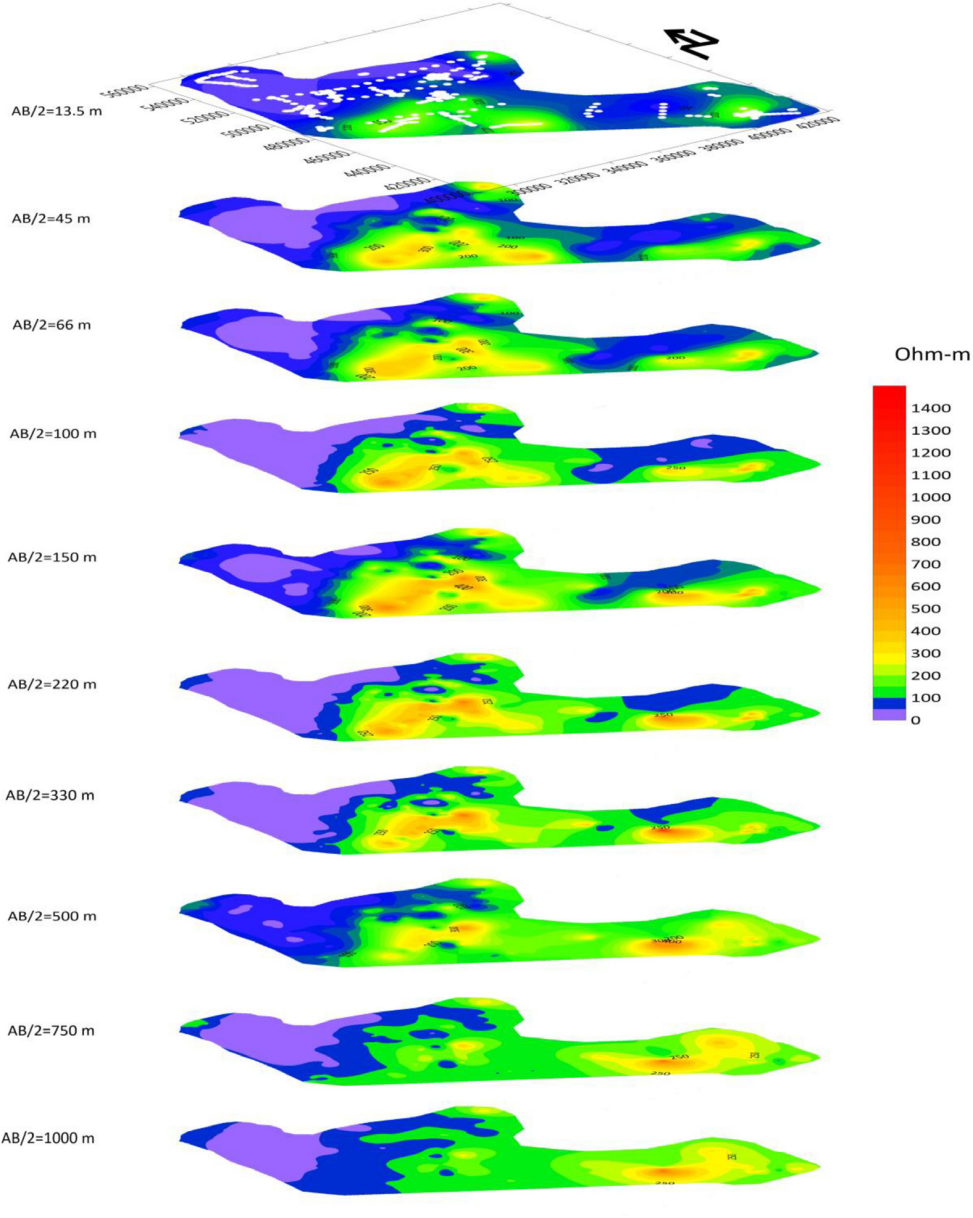
Stacked section of apparent resistivity at different AB/2.

**Fig. 4:**
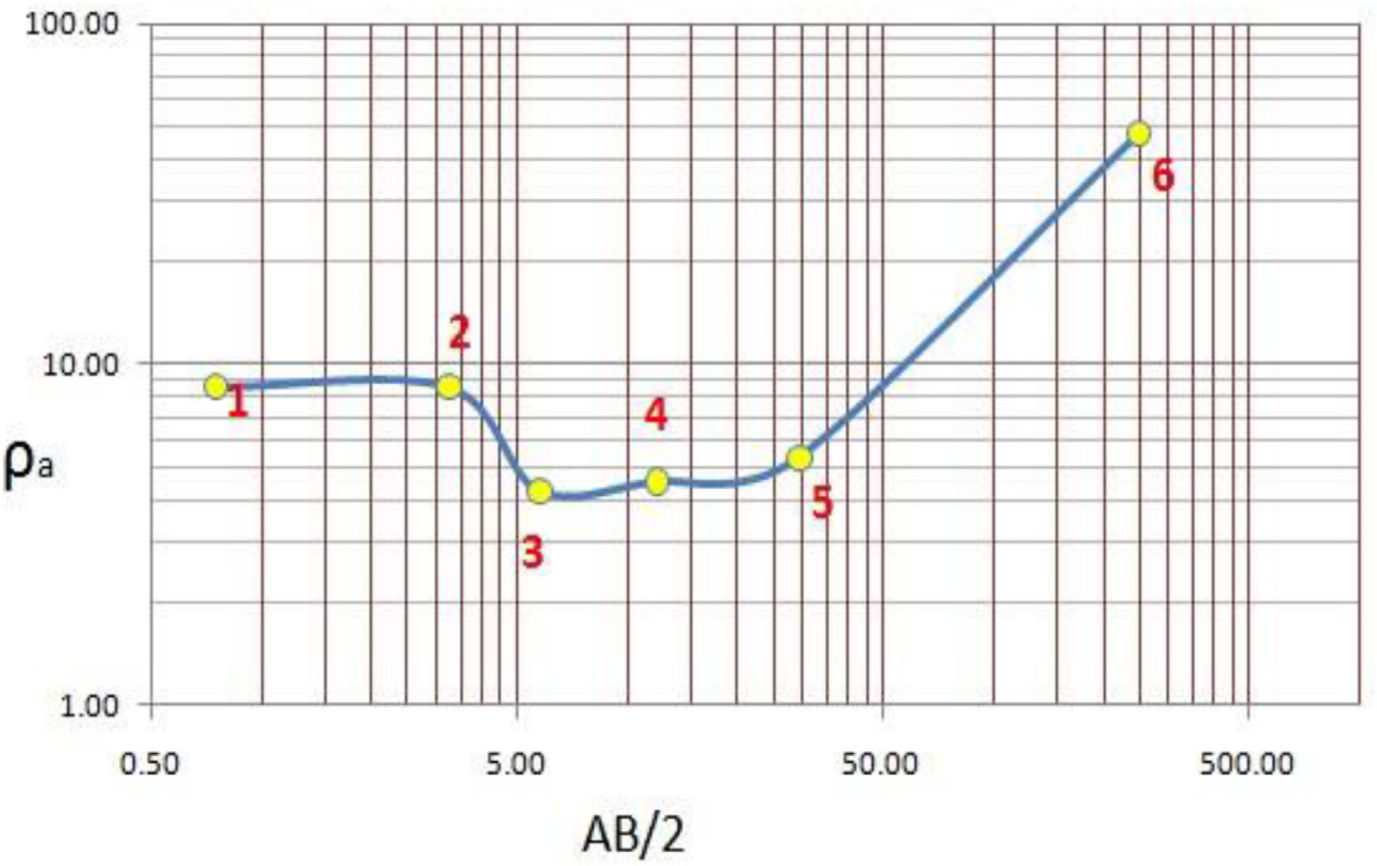
VES V-112 show how the characteristic points are determined. Numbers in red indicate inflection and extreme points.

About 288 Vertical electrical Sounding (VES) data using Schlumberger configuration with maximum half current electrode spacing (AB/2=1000m) were collected in several phases owned by Oromia water works design and supervision enterprise (OWWDSE). Table (1) summarizes the resistivity data collected in the Borena area. Apparent resistivity data at various AB/2 is presented in a stacked section in Fig. (3). And Fig. (4) and Table (2) provide CPs and derived parameters at VES (V-112) of the study area. The mean, median and standard deviation variation shows that the data need transformation. Thus, logarithmic transformation is used before modeling variogram, which would also help us to remove negative values.

**Table 1:** Summary of VES data collected

Total VES	Total *ρ_a_* data	min	max	mean	median	Standard Dev
288	5182	0.2	2400	132.2	52	188

**Fig. 5:**
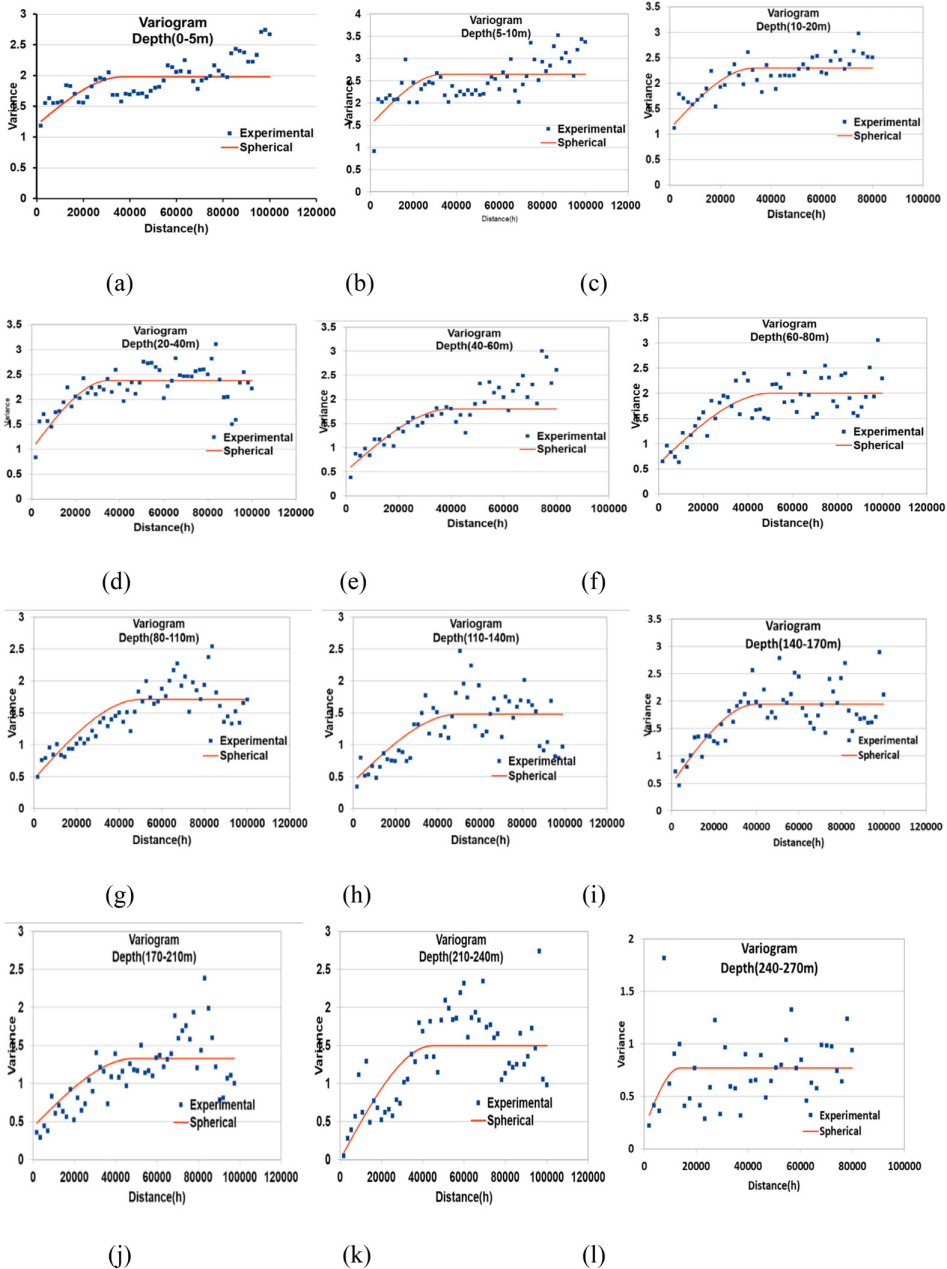

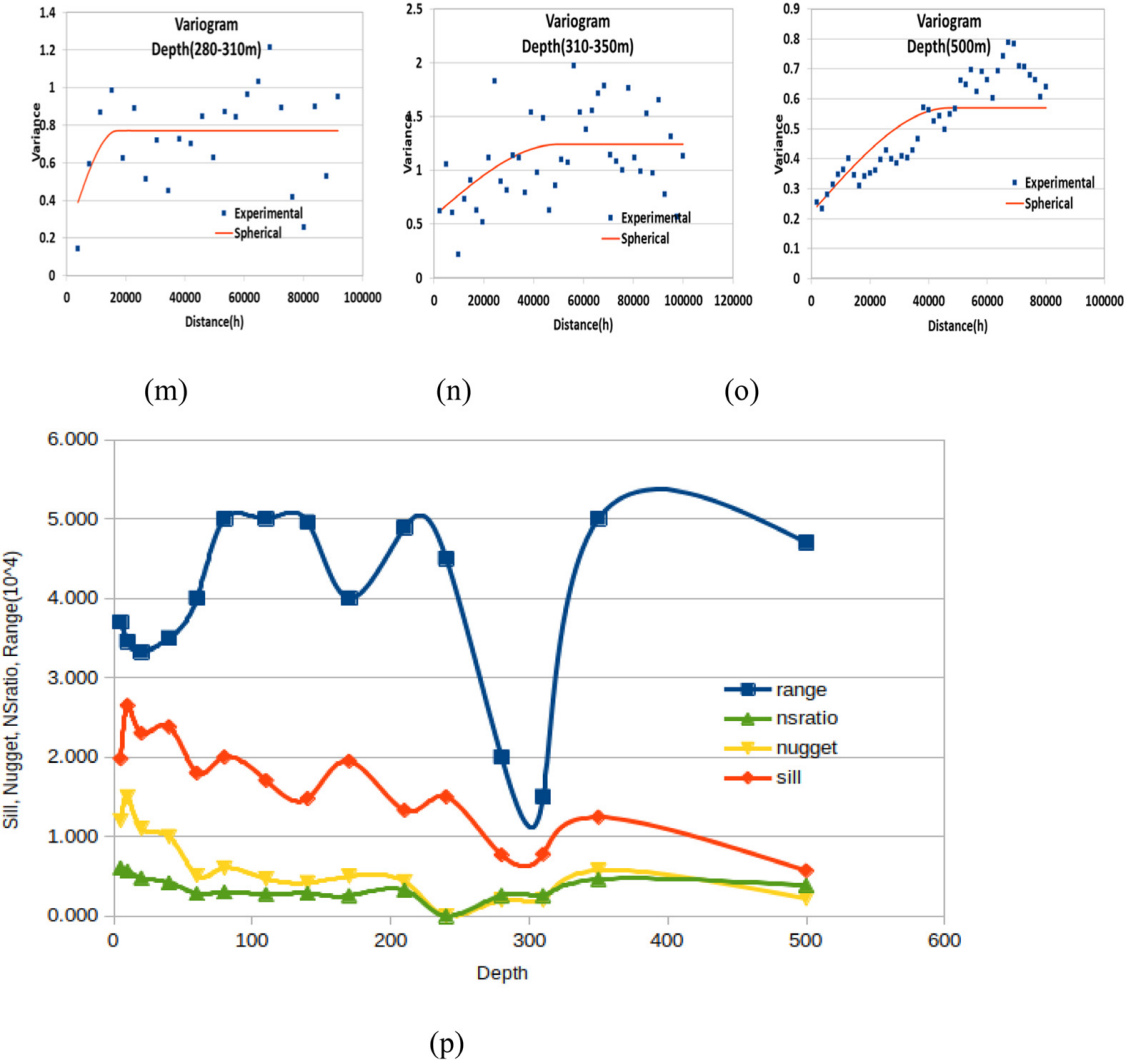
Variograms for various depth ranges (a-o) and Correlation of Nugget, Sill and Range (p), respectively.

**Table 2:** Depths and Resistivies determined from the CPs of VES V-112

CP no	Depth(m)	*ρ_a_*(*Ohm − m*)
1	0.75	8.57
2	3.26	8.75
3	5.76	4.26
4	12.02	4.53
5	29.56	5.33
6	250	47.09

**Table 3:** Model variograms for different depths

Depth Range	Model Variogram	Nugget	Sill	Range(m)	Nugget/Sill	Spatial correlation
0-5	Spherical	1.20	1.98	37000	0.61	Moderate
5-10	Spherical	1.50	2.65	34545	0.57	Moderate
10-20	Spherical	1.10	2.30	33232	0.48	Moderate
20-40	Spherical	1.00	2.38	35000	0.42	Moderate
40-60	Spherical	0.51	1.80	40000	0.28	High
60-80	Spherical	0.60	2.00	49991	0.30	High
80-110	Spherical	0.47	1.71	50030	0.27	High
110-140	Spherical	0.42	1.48	49593	0.29	High
140-170	Spherical	0.50	1.95	40000	0.26	High
170-210	Spherical	0.43	1.33	48892	0.33	High
210-240	Spherical	0.00	1.5	45000	0.00	High
240-270	Spherical	0.20	0.77	20000	0.26	High
280-310	Spherical	0.20	0.78	15046	0.26	High
310-350	Spherical	0.57	1.24	50042	0.46	Moderate
500	Spherical	0.22	0.57	47000	0.39	High

### Iso-apparent resistivity maps

Qualitative interpretation of VES data provides an overview of the distribution of resistivity variation over the study area. The qualitative interpretation procedure involves analysis of curve types, construction of apparent resistivity maps, combined traversing/profiling approaches. The Iso-apparent resistivity maps show lateral apparent resistivity variations with a function of pseudo-depth (AB/2 as depth). These maps show the general lateral variation of resistivity in the area under study. In this study, Iso-apparent resistivity maps for various depths (AB/2= 13.5, 45, 66, 100, 150, 220, 330, 500, 750 and 1000m) were constructed. The choices of depths are based on the variations it shows and the interest in corresponding expected depths. The first work in qualitative analysis of a range of VES data is to plot the apparent resistivity distribution map. This would provide information on the heterogeneous nature of the subsurface. Stacked plan maps of sliced depth sections are a convenient way to look at resistivity distribution in the vertical and lateral directions. Fig. 3 shows a stacked plot of apparent resistivity distribution on various sliced depth sections. The geostatistical interpolation methods (Ordinary Kriging) have been applied to construct two-dimensional maps.

The distribution of the measured apparent resistivity indicates that the area is electrically heterogeneous. Generally, the area can be grouped into two resistivity zones. The Northern region typically have a low resistive horizon, and the middle to the eastern region show relatively high resistive zones in all depth maps. Patches of higher resistivity regions are shown in the middle of the study area. The low resistivity region dominates most of the area at pseudo-depth from 13.5 to 66 m of half electrode spacing. Whereas, at pseudo-depth maps from AB/2=100 - 500 m, medium resistivity regions dominate the study area. The medium resistivity region continues dominating below this depth however shows a relative decrease in resistivity value.

Variograms are modeled using SAGA GIS [5], an open-source gis platform. By modeling variograms kriging interpolation is used to prepare 2D maps for different depths..

## Discussion

**Fig. 6:**
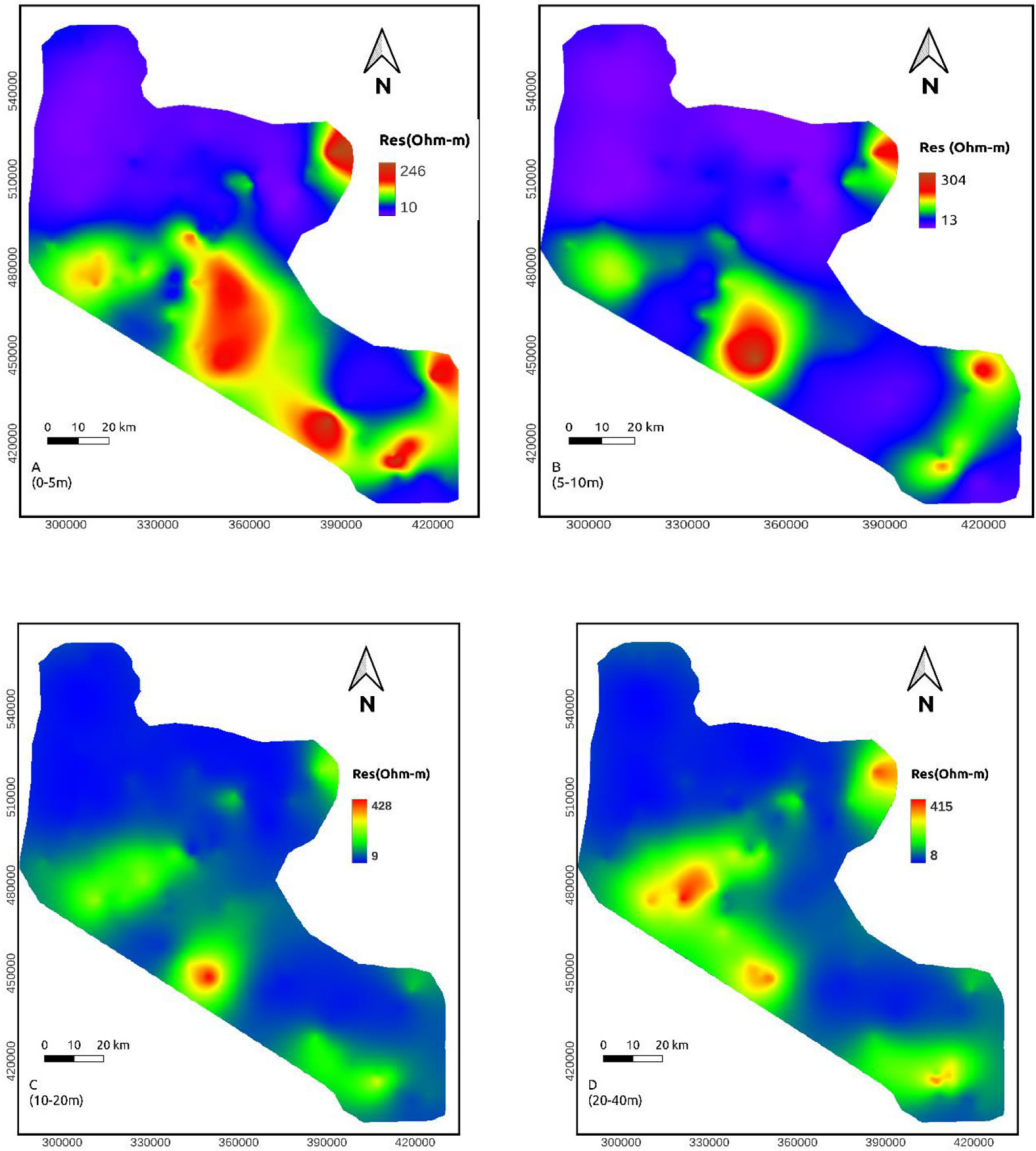

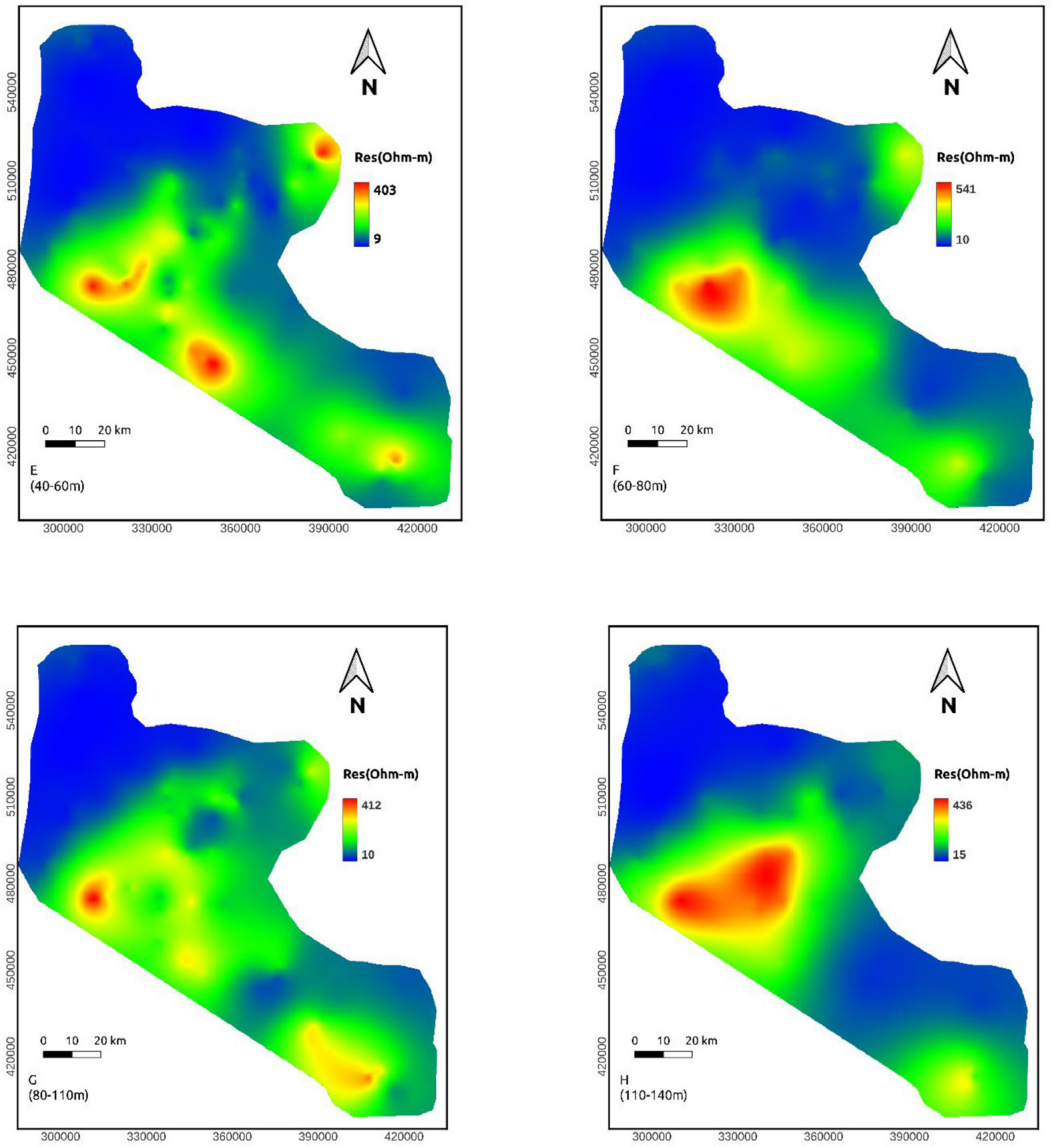

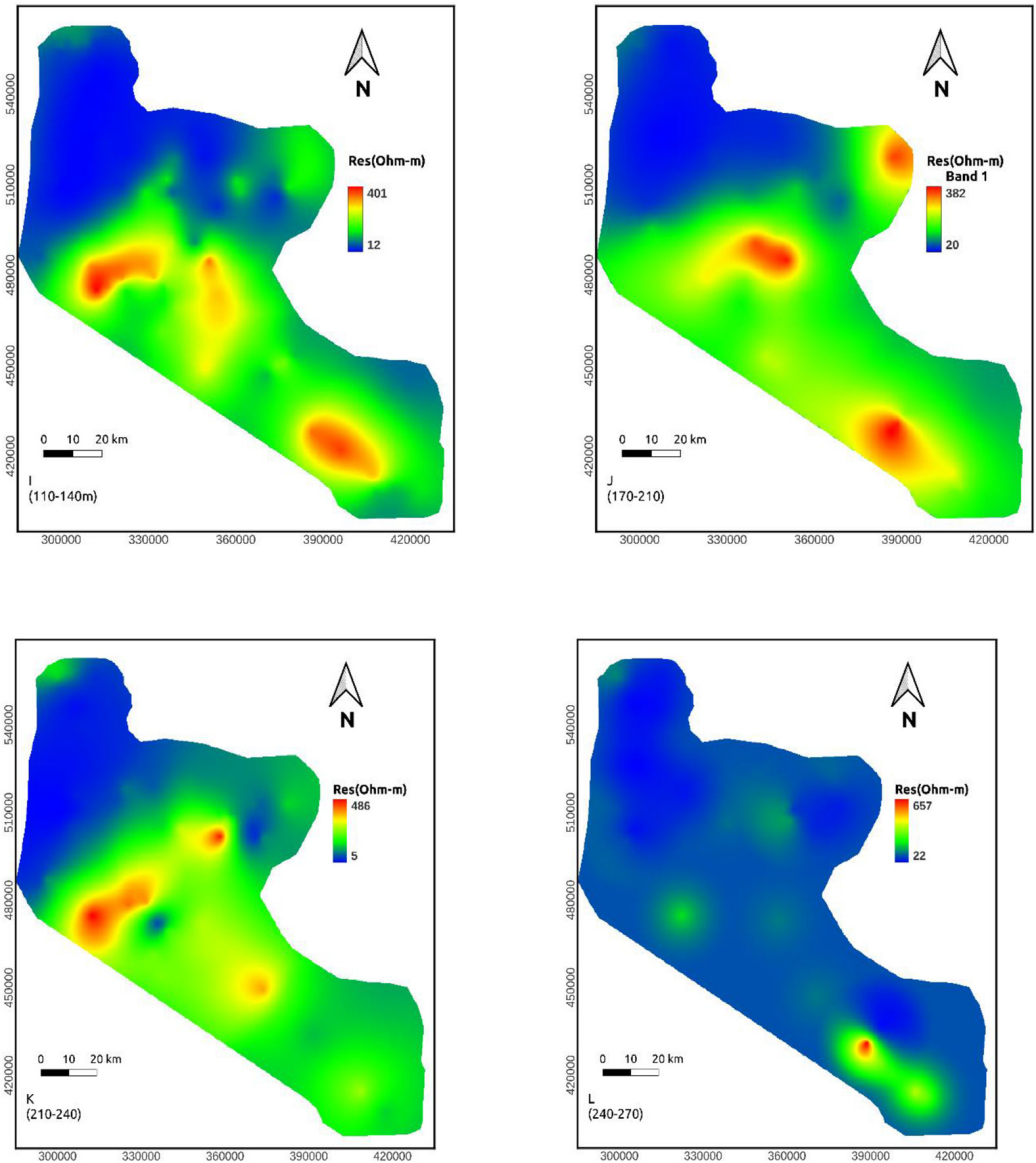

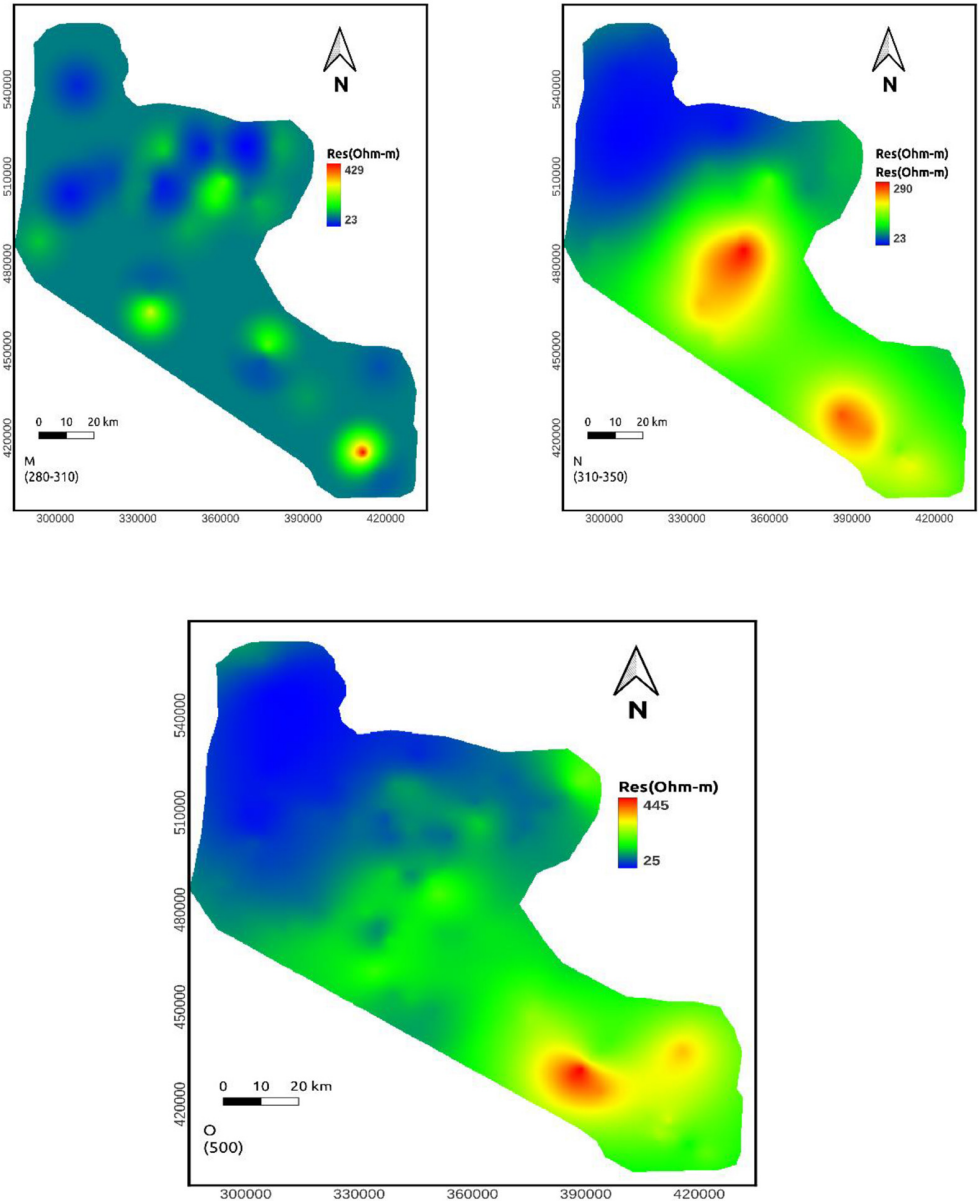
Resistivity map obtained from the approximate mapping of VES data for various depth ranges in meter (A. 0-5 m, B. 5-10 m, C, 10-20m, D, 20-40, E. 40-60m, F. 60-80m, G. 80-110m, H.110-140m, I. 140-170m, J. 170-210m, K. 210-240m, L. 240-170m, M. 280-310m, N. 310-350, O. 500m).

**Depth maps of (0-5, 5-10 and 10-20m)**: The resistivity values at these depths range from 10-248, 11-304 and 9-428 Ohm-m, respectively. In these maps, the minimum resistivity showed on northern side of the area. The maximum resistivity values are distributed at the middle and SE flank of the study area. The resistivity shows a decrease down depth, reflecting relatively resistive shallow subsurface as a response to dry soil (Fig. 6a-c).

**Depth maps (20-40, 40-60, 60-80, and 80-110m)**: The resistivity values of this depth range vary from 8-416, 9-403, 10-541 and 11-413 Ohm-m, respectively. Compared to the above depth ranges, these maps show more resistive region in the middle part (Fig. 6d-g).

**Depth maps (110-140, 140-170, 170-210, and 210-240m)**: The resistivity value at these depths lie within a range of 5-486 ohm-m. Still, the northern part of the area shows relatively conductive rock. At the same time, the middle sections are relatively resistive (Fig. 6h-6k).

**Depth maps (240-270 and 280-310m)**: The resistivity value at these depths lie within a range of 22-657 ohm-m. A vast portion of these maps shows low resistivity values except for some small patches of high resistivity in the middle and SE part of the study area. This extensive section may be favorable to groundwater investigation (Fig. 6l-m). High resistivity values the central and SE part of the study area reappeared at the remaining depth maps. However, the low resistivity portion of the northern part of the study area continues to a deeper depth (Fig. 6n-o).

Resistivity distribution from the approximate mapping of VES data shows that low resistivity values are recorded to the northern part of the study area in all depth range whereas the middle part of the study area shows high resistivity in most depth maps.

## Conclusion

About 288 resistivity sounding data points (VES) with Schlumberger configuration were used to map potential groundwater zones. Without the explicit assumption of the true resistivity distribution, the subsurface can be effectively mapped using characteristics points obtained from inflection and extreme points of the VES curve and geostatistical interpolation. The characteristics points are determined from each VES data. The variograms were modelled in SAGA GIS to determine the weight used in the kriging interpolation method. The technique was implemented to map the 3D distribution of 288 VES data in the Borena area. Apparent resistivity measurements, collected using Schlumberger array, range from 0.2 ohm-m to 2400 ohm-m with a mean value of 130.5 ohm-m. The study aimed to assess near-surface electrical resistivity data distribution acquired at the Borena area and to determine potential groundwater zones. Resistivity responses of various deposits in the study area were mapped. Very low to low resistivity variations are mapped in the northern end of the study area, while medium to the moderately resistive ground is mapped in the middle and southern part of the study area. The low resistivity horizon at the shallow subsurface may be due to saline rocks since the area has numerous saline craters and maars.

Automatic mapping of VES data with geostatistical treatment has facilitated the interpretation and provided a sound picture of the subsurface geology in terms of resistivity distribution. By integrating additional geophysical, geochemical and remote sensing data, the methodology can be used to precisely identify potential groundwater zone in a regional scale using GIS and multi-criteria decision methods.

## Data availability

Data will be made available on request.

## Declaration of Competing Interest

The authors declare that there is no conflict of interest.
